# Conservative plausibility-filtered SMOTE for credit card fraud detection under extreme class imbalance

**DOI:** 10.3389/frai.2026.1871972

**Published:** 2026-07-17

**Authors:** Fray L. Becerra-Suarez, Paolo P. Arones-Perez, Frederik F. Zamora-Del-Aguila, Manuel G. Forero

**Affiliations:** 1 Grupo de Investigación en Inteligencia Artificial (UMA-AI), Facultad de Ingeniería, Universidad Privada Norbert Wiener, Lima, Peru; 2 Semillero Lún, Universidad de Ibagué, Ibague, Colombia

**Keywords:** credit card fraud detection, data augmentation, extreme class imbalance, oversampling, SMOTE, supervised plausibility, tabular learning

## Abstract

Detecting credit card fraud is a complex tabular learning problem due to the underrepresentation of fraudulent events, extreme class imbalance, and scattered minority areas. To address this limitation, a conservative minority synthesis strategy is proposed, combining the removal of minority outliers, synthetic data generation using SMOTE, geometric filtering, and supervised plausibility filtering. Unlike conventional oversampling, the method does not seek to artificially balance classes but rather to retain only locally consistent and discriminatively useful synthetic samples. The approach was evaluated on the ULB Credit Card Fraud Detection and IEEE-CIS Fraud Detection datasets using a 60/20/20 stratified partitioning, preventing information leakage between training, calibration, and testing. On ULB, the proposal retained 638 final synthetic samples, raising the fraud rate of the augmented dataset to 0.005396. In IEEE-CIS, 947 final synthetic samples were retained, with a final fraud rate of 0.037563. Compared to techniques such as SMOTE, Borderline-SMOTE, ADASYN, and SimpleVAE, the proposed method showed competitive performance, achieving the highest AUPRC in four of six classifiers in ULB and in three of six classifiers in IEEE-CIS. Ablation analyses and statistical validation show that supervised plausibility filtering helped control the quality of the generated samples, although its benefit is not uniform and depends on the classifier and dataset used.

## 1 Introduction

In recent years, the financial ecosystem has undergone a profound transformation in the way people make payments, purchases, credit applications, and other transactions through internet-connected platforms. This expansion of the transactional infrastructure has enhanced the availability, speed, and efficiency of financial services; however, it has also broadened the attack surface for fraudulent practices that exploit the speed, scale, and heterogeneity of contemporary payment systems to execute increasingly dynamic and difficult-to-detect schemes (Theodorakopoulos et al., 2025). In this context, automated credit card fraud detection can no longer be viewed solely as a mechanism for containing financial losses. Its role also extends to preserving user trust, operational continuity, and the institutional capacity to respond to risks inherent in digital financial services (Mienye and Jere, 2024; Breskuviene and Dzemyda, 2024; Lescano-Delgado, 2023).

It should be noted that the difficulty of the problem does not lie solely in selecting a classifier with greater predictive power. In real-world scenarios, detection systems operate on heterogeneous tabular data, with an extreme imbalance between classes, a very low frequency of fraudulent events, and attack patterns scattered across the feature space. Unlike other classification problems, fraud rarely forms a compact, homogeneous, and clearly separable region. Rather, it typically manifests as small, sparsely represented local clusters close to legitimate transactions, which makes their statistical and operational delineation difficult (Baisholan et al., 2025; Cherif et al., 2023; Hilal et al., 2022). Under these conditions, fraud detection should not be treated as a conventional binary classification problem, but rather as a highly imbalanced supervised learning problem with high operational sensitivity, where the low prevalence of the positive class limits the models' ability to learn stable, generalizable, and representative decision boundaries (Becerra-Suarez et al., 2025a).

In light of this limitation, data augmentation has been used as a strategy to enrich the representation of the minority class during training. Classic oversampling methods, particularly SMOTE and some of its variants, continue to be used due to their simple implementation, low computational cost, and ability to improve the sensitivity of classifiers in financial applications (Gupta et al., 2025; Mia et al., 2025). Their usefulness, however, depends on a geometric assumption that does not always hold in fraud domains. By generating new observations through local interpolation among minority neighbors, these procedures do not explicitly verify whether the synthetic instances remain within plausible regions of the fraud manifold or whether they unduly approach areas dominated by the majority class. In financial contexts with severe imbalance, a non-restrictive synthetic generation can introduce ambiguous, locally inconsistent, or insufficiently informative samples, with the risk of deteriorating the decision boundary rather than strengthening it.

Several recent studies have shown that performance in extreme imbalance scenarios depends not only on the final classifier but also on how the synthetic samples are generated and selected. Strategies based on controlled Gaussian noise have been proposed, as well as comparisons between SMOTE, ADASYN, and GNUS in combination with models such as Random Forest and XGBoost, demonstrating that the usefulness of augmentation varies according to the degree of imbalance, the structure of the dataset, and the predictive algorithm used (Imani et al., 2025; Becerra-Suarez et al., 2025b). However, these approaches still have significant limitations: their performance is not always consistent across models, they may incorporate artificial samples that are not very discriminating, and in many cases, they do not explicitly control the preservation of the decision boundary or the local plausibility of the generated examples.

A different line of research has examined more expressive generative mechanisms, including tabular GANs, variational autoencoders, recurrent hybrid models, and sequential approaches sensitive to temporal patterns (Mienye and Swart, 2024; Tayebi and El Kafhali, 2025; Wang and Nguyen, 2025). Yousefimehr and Ghatee (2025) emphasized that the value of synthetic data is not determined only by its volume. Its utility also depends on whether the generated samples preserve the minority distribution and remain coherent with the sequential dynamics of fraudulent transactions, particularly when augmentation is combined with hybrid architectures such as LightGBM-LSTM. Along similar lines, (Lee and Bae, 2025) reported that variational-autoencoder-based augmentation can improve classification on imbalanced tabular data when integrated into deep ensemble architectures, although the magnitude of this improvement remains dependent on the dataset and on the predictive model used afterward.

Furthermore, comparative studies of generative methods have shown that there is no universally superior synthesis technique for unbalanced tabular data. Won et al. (2026), comparing SMOTE, Gaussian Copula, TVAE, and CTGAN, reported that the performance of each method depends on the level of imbalance and the specific characteristics of the problem. Similarly, Kindji et al. (2025) demonstrated that tabular generative models are highly sensitive to preprocessing, feature coding, and hyperparameter tuning. These findings reinforce the need for conservative approaches that do not simply generate a larger number of examples but rather incorporate explicit criteria for selecting those artificial samples that are plausible, locally consistent, and discriminatively useful for fraud detection.

A representative example of this trend is AGSS, Adaptive Generative Synthetic Sampling, an approach that combines density-based clustering using DBSCAN with synthetic generation restricted to dense minority clusters, excluding scattered or noisy points before the interpolation process (Nama and Banu, 2026). Furthermore, AGSS introduces a curvature perturbation aimed at increasing the diversity of the artificial samples without losing local geometric consistency. According to its authors, this mechanism reduces overlap between classes, improves adaptation to complex distributions, and limits overfitting compared to methods such as SMOTE, Borderline-SMOTE, and KMeans-SMOTE. This type of approach is relevant because it confirms that, in severely unbalanced scenarios, augmentation should not be conceived as a quantitative expansion of the minority class, but rather as a structural reinforcement process guided by dense, reliable, and representative regions.

From this perspective, the central challenge lies not only in synthesizing more fraudulent transactions, but also in identifying which artificial examples should be incorporated into the training to strengthen the decision boundary without introducing synthetic noise. Based on this, the present work proposes a conservative minority synthesis strategy for detecting credit card fraud under extreme imbalance. The method uses SMOTE as the base generation mechanism but restricts the incorporation of synthetic samples through two successive controls: a geometric filter based on neighborhoods of the fraudulent class and a supervised plausibility filter using an auxiliary classifier trained exclusively on real data from the training set. This methodological decision seeks to preserve the operational simplicity of SMOTE while introducing an explicit control mechanism over the quality and discriminative utility of the generated samples.

The main contributions of this study are threefold. First, a conservative augmentation scheme is proposed that shifts the emphasis from massive rebalancing to the discriminative selection of synthetic samples from the minority class. Second, a sequential, geometric, double-filtering mechanism is integrated to discard isolated, locally inconsistent, or insufficiently plausible synthetic samples for the fraudulent class. Third, an experimental protocol is established with strict separation between training, calibration, and test sets, complemented by an ablation analysis that allows estimation of the incremental contribution of each component of the proposed method.

This work is justified on three levels. Theoretically, it contributes to modeling fraud as a localized and contextual anomalous signal, closer to a pattern of structured deviation than to a homogeneous rare class. Methodologically, it shifts the discussion from the number of synthetic samples to their actual discriminatory utility, emphasizing the need to evaluate augmentation based on its effect on the decision frontier. Finally, practically, it offers a scalable and operationally sound alternative for fraud detection systems, where the reduction of false negatives must be carefully balanced with the control of false positives, due to its implications for review costs, customer friction, and the institutional efficiency of digital financial services.

## 2 Materials and methods

### 2.1 Materials

Two datasets were used for this research. The ULB Credit Card Fraud Detection (ULB) dataset was chosen because it is widely used as a reference in research on financial fraud detection and supervised learning (ML) with extremely unbalanced classes. It contains records of credit card transactions made by European cardholders during September 2013 (Le, 2021). It comprises a total of 284,807 records, of which 492 correspond to fraudulent transactions (approximately 0.173% of the total records). Furthermore, it includes 31 descriptors, of which the target descriptor, Class, is coded with the value 0 to represent legitimate transactions and the value 1 for fraudulent transactions. The other descriptors are anonymized and defined from V1 to V28, including the variables Time and Amount.

The IEEE-CIS Fraud Detection (IEEE-CIS) dataset contains 590,540 transactions, of which 20,663 are fraudulent, representing approximately 3.5% of the total (Kabartay, n.d.). It includes a total of 202 descriptors. The target descriptor is defined by the variable is Fraud, while the predictor variables include transactional information, card-related attributes, product characteristics, device information, and anonymized identity descriptors. Among the most relevant descriptors are TransactionAmt, which records the transaction amount, and TransactionDT, which indicates the relative elapsed time from a reference point. Although the fraud prevalence is higher than in ULB, IEEE-CIS still presents a markedly imbalanced distribution, making it suitable for assessing augmentation procedures, resampling schemes, and evaluation metrics oriented toward fraud detection.

The tests of the augmentation methods and ML classifiers were performed using a computer with an Intel Core Ultra 9 275HX processor, 32GB RAM, Windows 11 64-bit operating system and an NVIDIA GeForce RTX 5080 GPU. On this working environment, the different activities were executed from data preprocessing, generation of synthetic samples, training of the classifiers, calibration of probabilities and the comparative evaluation of the models.

### 2.2 Data processing and partitioning

Based on the general characterization of the datasets described in the previous section, an exploratory analysis was conducted to characterize the dataset structure and verify its initial quality conditions. In both cases, the variables associated with the transaction amount and time were examined due to their relevance in describing transactional behavior and the potential presence of differences between legitimate and fraudulent transactions. To improve the visual interpretation of the amounts, the logarithmic transformation log(1 + Amount) was applied. In the ULB dataset, the variables Amount and Time were analyzed, while in the IEEE-CIS dataset, their equivalents TransactionAmt and TransactionDT were used.

**Figure 1:**
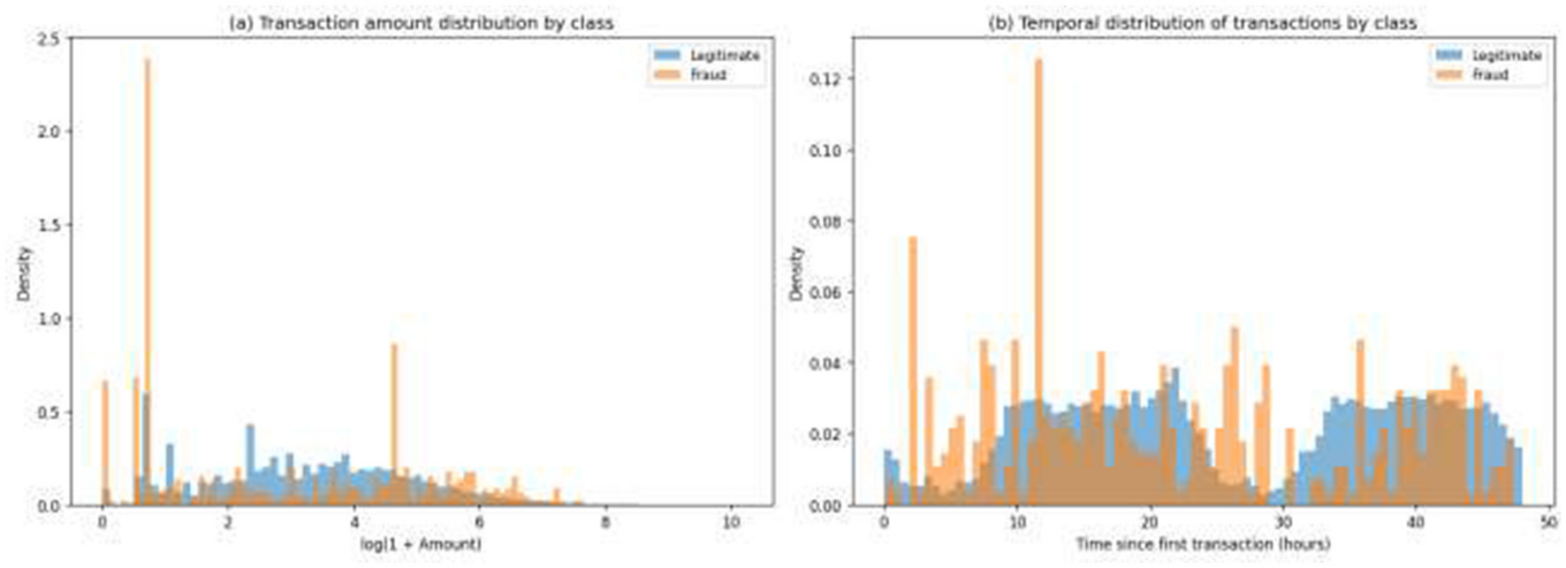
Exploratory distribution of transaction amounts and temporal behavior by class in the ULB dataset.

As shown in Figure 1, the amounts of legitimate and fraudulent transactions do not follow exactly the same pattern, but neither are they clearly separated (panel a). In several ranges, there is significant overlap between the two classes, which is not enough to distinguish a fraudulent transaction from a legitimate one. Even so, this variable provides useful information about how financial transactions are distributed. Regarding the Time variable (panel b), fraudulent transactions are not concentrated at a single point in time, but are distributed throughout the analyzed period, with some intervals where their presence is more noticeable.

**Figure 2:**
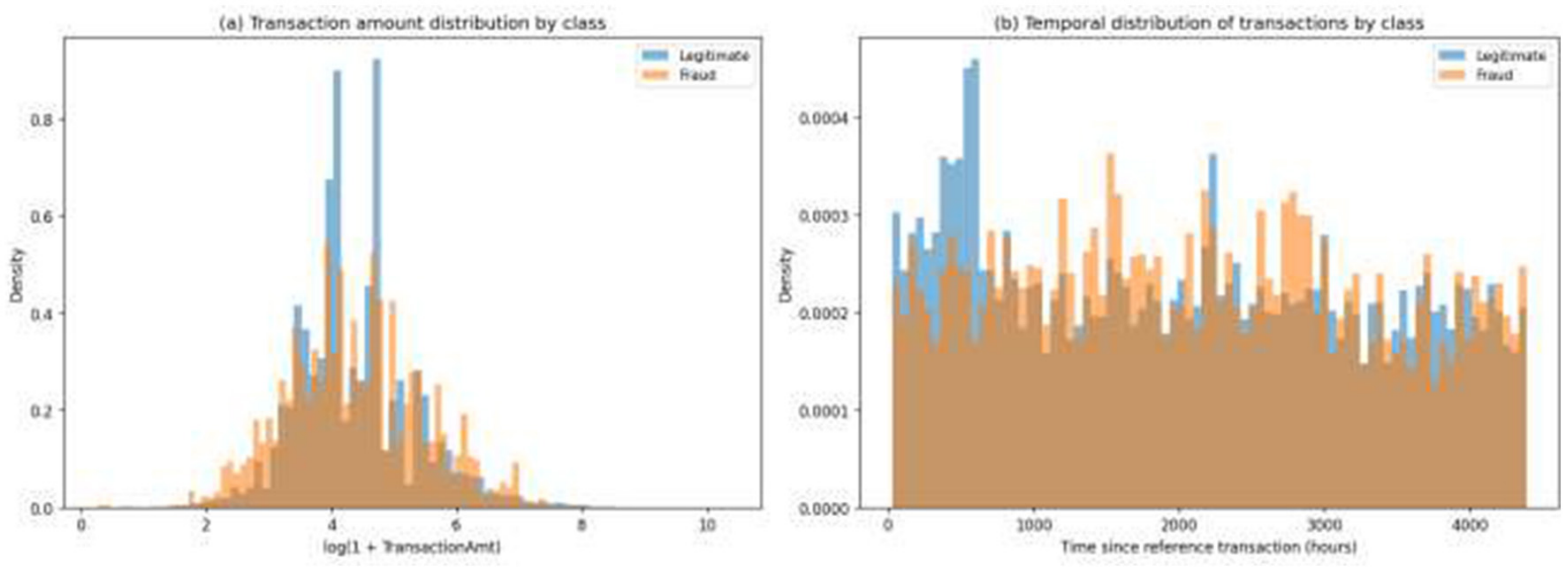
Exploratory distribution of transaction amount and temporal behavior by class in the IEEE-CIS dataset.

In Figure 2, the TransactionAmt variable also showed an asymmetric distribution with overlap between legitimate and fraudulent transactions, even after applying the logarithmic transformation. The TransactionDT variable, expressed in hours from a reference date, shows a more extensive temporal distribution than in the ULB set and reveals that fraudulent transactions are dispersed throughout the observation period. Both analyses demonstrated that fraud does not appear as a simple, compact pattern or easily separable by individual variables, but rather as a minority and dispersed behavior within the transactional space.

In addition to visual analysis, a data quality assessment was performed before partitioning the dataset. In ULB, no infinite values (positive or negative) were identified in the numerical variables. Likewise, no categorical predictors or descriptors with the same value in all records were found. However, 1,081 duplicate records were identified and removed from the original dataset. After this cleaning, 283,253 legitimate transactions and 473 fraudulent transactions were retained, corresponding to a fraud rate of approximately 0.167%. The same review criteria were applied to IEEE-CIS. No duplicate records or infinite values were identified that would justify removal under the defined criteria. The missing values were retained and subsequently processed using median imputation within the preprocessing pipeline.

To prevent information leakage, both datasets were partitioned before any preprocessing, calibration, or augmentation stages. In both cases, stratified sampling was applied to three mutually exclusive subsets: 60% for training, 20% for calibration, and 20% for final testing, preserving the original proportion of the fraudulent class in each partition. In the ULB dataset, this division yielded 170,883 transactions for training, 56,962 for calibration, and 56,962 for testing. In the IEEE-CIS dataset, 354,324 transactions were obtained for training, 118,108 for calibration, and 118,108 for testing.

The preprocessing consisted of median imputation and z-score standardization, adjusted exclusively with the training subset and then applied to calibration and testing. In IEEE-CIS, this pipeline was applied to the previously defined numerical representation. Thus, the transformation parameters did not introduce external information into the training. All partitions were performed with a fixed random seed of 42.

### 2.3 Proposed method

Standard oversampling techniques, such as SMOTE and its variants, rely on the assumption that the minority class can be strengthened through local interpolation between nearby real observations. Operationally, these techniques generate new instances along the feature space segments connecting neighboring minority samples. However, in domains with extreme imbalance, this generation can be problematic, as it fails to verify whether the artificial examples remain within plausible regions of the fraudulent distribution or whether they unduly approach areas dominated by the majority class. Consequently, uncontrolled oversampling can populate ambiguous regions of the decision boundary, amplify noisy minority patterns, or introduce uninformative observations, risking classifier performance degradation and distorting learned margins.

The proposed method is therefore conceived as a selective and conservative extension of SMOTE, rather than as a separate generative model. SMOTE is maintained as the base synthesis mechanism because of its simplicity, reproducibility, and broad adoption in imbalanced learning. The methodological contribution lies elsewhere: not in increasing the number of synthetic observations, but in regulating their admission into the training set. For this purpose, the procedure incorporates explicit geometric and supervised plausibility controls, with the aim of reducing synthetic noise and retaining only those artificial samples that can contribute to the discrimination of fraudulent transactions.

Each stage of the procedure responds to a specific source of risk associated with oversampling under extreme imbalance. First, outlier removal within the fraudulent class decreases the probability of interpolating from isolated, unstable, or potentially noisy minority observations. Second, conservative SMOTE-based generation increases the representation of the minority class without forcing an artificial balance between classes, thereby limiting excessive distortion of the empirical distribution. Third, geometric filtering discards synthetic candidates that are weakly aligned with the local structure of actual fraudulent transactions. Finally, supervised plausibility filtering eliminates candidates that, while geometrically close, are not recognized as sufficiently compatible with fraud by an auxiliary classifier trained exclusively on real data from the training set.

The entire procedure was developed exclusively on the training set (*D*_*train*_). To facilitate understanding and reproducibility of the method, the complete procedure is summarized in Algorithm 1. This pseudocode shows the operational sequence of the proposed approach: debugging of minority outliers, conservative generation using SMOTE, geometric filtering of minority coherence, and supervised plausibility filtering.

Algorithm 1 Pseudocode of the conservative plausibility-filtered SMOTE method. 
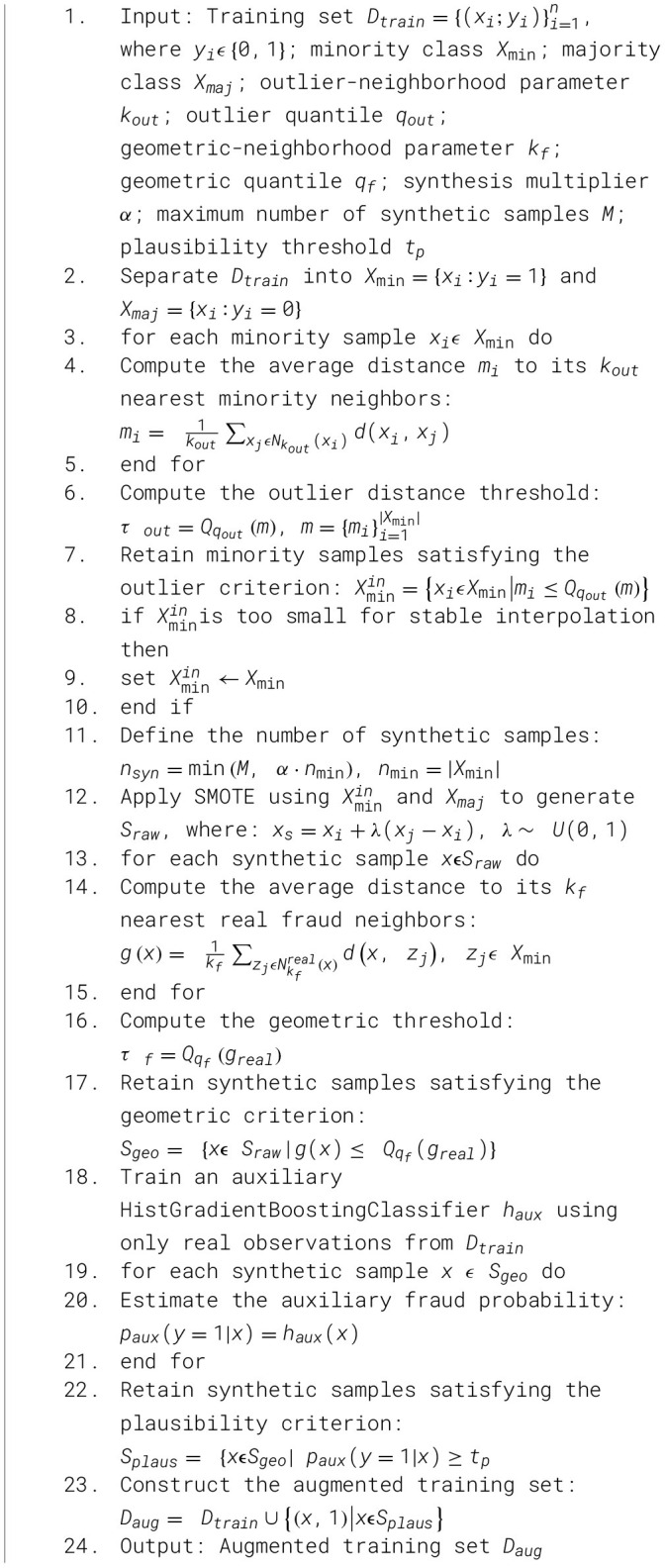

Starting with Algorithm 1, the following subsections describe in greater detail each of the phases that make up the proposed method. First, the removal of minor outliers is presented, aimed at preventing interpolations from isolated fraudulent observations. Then, conservative synthetic generation using SMOTE is described, followed by geometric filtering for minor coherence and supervised plausibility filtering.

#### 2.3.1 Removal of minority outliers

First, the minority class was cleaned to reduce the risk of SMOTE interpolating from excessively isolated frauds. For each minority sample *x*_*i*_ϵ* X*_min_, its average distance to its *k*_*out*_ v nearest minority neighbors was calculated using Equation 1:


$$\begin{array}{l} m_{i}=\;\frac{1}{k_{out}}\underset{x_{j}\epsilon N_{k_{out}}\left(x_{i}\right)}{\sum }{d(x}_{i},x_{j}), \end{array}$$ (1)

where $N_{k_{out}}^{\min}\;\left(x_{i}\right)$ represents the set of *k*_*out*_ nearest neighbors of *x*_*i*_ within the actual minority class *X*_min_ and *d*(·, ·) denotes the distance used in the feature space. Starting from the distribution of mean distances defined as $m={\left\{m_{i}\right\}}_{i=1}^{\left|X_{\min}\right|}$, the outlier removal threshold was defined according to Equation 2:


$$\begin{array}{l} {\tau \;}_{out}=Q_{q_{out}}\left(m\right) \end{array}$$ (2)

where *Q*_*q*_*out*__(·) represents the selected removal quantile. Subsequently, only the minority observations whose mean distance did not exceed this threshold were obtained using Equation 3:


$$\begin{array}{l} X_{\min}^{in}=\left\{x_{i}\epsilon X_{\min}|m_{i}\leq Q_{q_{out}}\left(m\right)\right\} \end{array}$$ (3)

The subset $X_{\min}^{in}$ corresponds to the fraudulent observations retained as the basis for synthetic generation. If this subset proved too small to allow stable interpolation, the entire minority class *X*_min_ was used as an operational safety mechanism.

#### 2.3.2 Conservative synthetic generation using SMOTE

Starting with the refined minority $X_{\min}^{in}$ and the complete real majority *X*_*maj*_, SMOTE was applied to generate a bounded number of synthetic minority samples. Unlike conventional oversampling strategies, the goal was not to artificially equalize the number of legitimate and fraudulent transactions, but rather to selectively strengthen the representation of the minority class while preserving the highly unbalanced nature of the training set.

The maximum number of synthetic samples was defined according to Equation 4:


$$\begin{array}{l} n_{syn}=\;\min\left(M,\;\alpha \cdot n_{\min}\right),n_{\min}=|X_{\min}| \end{array}$$ (4)

where *n*_min_ represents the number of actual fraudulent observations in *D*_*train*_, α is a synthesis multiplier, and *M* is a maximum generation limit.

Interpolation was performed using the refined minority subset $X_{\min}^{in}$ as the basis. Operationally, each synthetic sample was generated by interpolation between a minority observation *x*_*i*_ and one of its minority neighbors *x*_*j*_, as expressed in Equation 5:


$$\begin{array}{l} x_{s}=x_{i}+\lambda \left(x_{j}-x_{i}\right),\;\lambda \sim \;U\left(0,1\right) \end{array}$$ (5)

The set of candidate synthetic samples generated at this stage was denoted in Equation 6 as:


$$\begin{array}{l} S_{raw}=\;{\left\{x_{s}\right\}}_{s=1}^{n_{syn}} \end{array}$$ (6)

This procedure allowed a controlled increase in the representation of the fraudulent class without imposing an aggressive rebalancing of the classes.

#### 2.3.3 Minority coherence geometric filtering

The generated synthetic samples were subjected to a geometric minority coherence filter. This step aimed to discard synthetic candidates that, although produced by SMOTE, were too far removed from the local structure of the actual fraudulent transactions.

For each synthetic sample *x ϵ S*_*raw*_, its average distance to its nearest real fraudulent neighbors *k*_*f*_ was calculated according to Equation 7:


$$\begin{array}{l} g\left(x\right)=\;\frac{1}{k_{f}}\underset{z_{j}\epsilon N_{k_{f}}^{real}\left(x\right)}{\sum }d\left(x,\;z_{j}\right),\;z_{j}\;\epsilon \;X_{min}, \end{array}$$ (7)

where $N_{k_{f}}^{real}\left(x\right)$ represents the set of the nearest real fraudulent neighbors *k*_*f*_ to the synthetic sample *x* and *z*_*j*_ denotes an actual fraudulent observation belonging to *X*_min_.

This value was compared with a reference obtained from the actual intra-minority average distances. Based on this distribution, the geometric threshold was defined as shown in Equation 8:


$$\begin{array}{l} {\tau \;}_{f}=Q_{q_{f}}\left(g_{real}\right), \end{array}$$ (8)

where *g*_*real*_ represents the distribution of mean distances between actual fraudulent observations and *Q*_*q*_*f*__(·) corresponds to the geometric quantile used as the threshold. Only synthetic samples that satisfied *S*_*geo*_ = {*xϵ S*_*raw*_|*g*(*x*) ≤ τ_*f*_} were retained. This step allowed for the discarding of floating synthetic examples or those that were poorly consistent with the local structure of the minority class.

#### 2.3.4 Plausibility filtering using an auxiliary classifier

Finally, the synthetic samples retained by the geometric filter were evaluated using a supervised plausibility filter. For this purpose, an auxiliary classifier *h*_*aux*_ implemented using HistGradientBoostingClassifier, was trained exclusively using real observations from the training set *D*_*train*_. This decision prevented the auxiliary classifier from learning from previously generated synthetic samples.

For each surviving synthetic sample *x ϵ S*_*geo*_, the auxiliary classifier estimated the probability of belonging to the fraudulent class, as defined in Equation 9:


$$\begin{array}{l} p_{aux}\left(y=1|x\right)=h_{aux}\left(x\right) \end{array}$$ (9)

Only those synthetics whose auxiliary probability was equal to or greater than the plausibility threshold *t*_*p*_ where retained. The final subset of plausible synthetics was defined as according to Equation 10:


$$\begin{array}{l} S_{plaus}=\;\{x\;\epsilon {\;S}_{geo}|\;p_{aux}(y=1|x)\geq t_{p} \end{array}$$ (10)

This filter discarded samples that, while geometrically close to the minority, were not considered sufficiently consistent from a discriminative point of view. Finally, the augmented training set was defined as shown in Equation 11:


$$\begin{array}{l} D_{aug}=\;D_{train}\cup \left\{\left(x,1\right)|x\epsilon S_{plaus}\right\}. \end{array}$$ (11)

In this expression, each synthetic sample *x ϵ S*_*plaus*_ is incorporated into the training set with label 1, corresponding to the fraudulent class. Therefore, *D*_*aug*_ represents the final set used to train the evaluated classifiers.

It is important to note that this plausibility filter was designed as a conservative quality control mechanism for the synthetic samples. By training only with real observations from *D*_*train*_, the auxiliary classifier avoids learning from previously generated synthetic samples and does not use information from the calibration or test subsets. However, this decision also implies that the filter tends to favor synthetic candidates compatible with fraudulent patterns already observed in the training set. Therefore, the method should be interpreted as a strategy aimed at preserving locally consistent and discriminatively plausible synthetic samples, not as a mechanism intended to discover new forms of fraud not represented in the available real data.

#### 2.3.5 Sequential visualization of the proposed method using t-SNE

**Figure 3:**
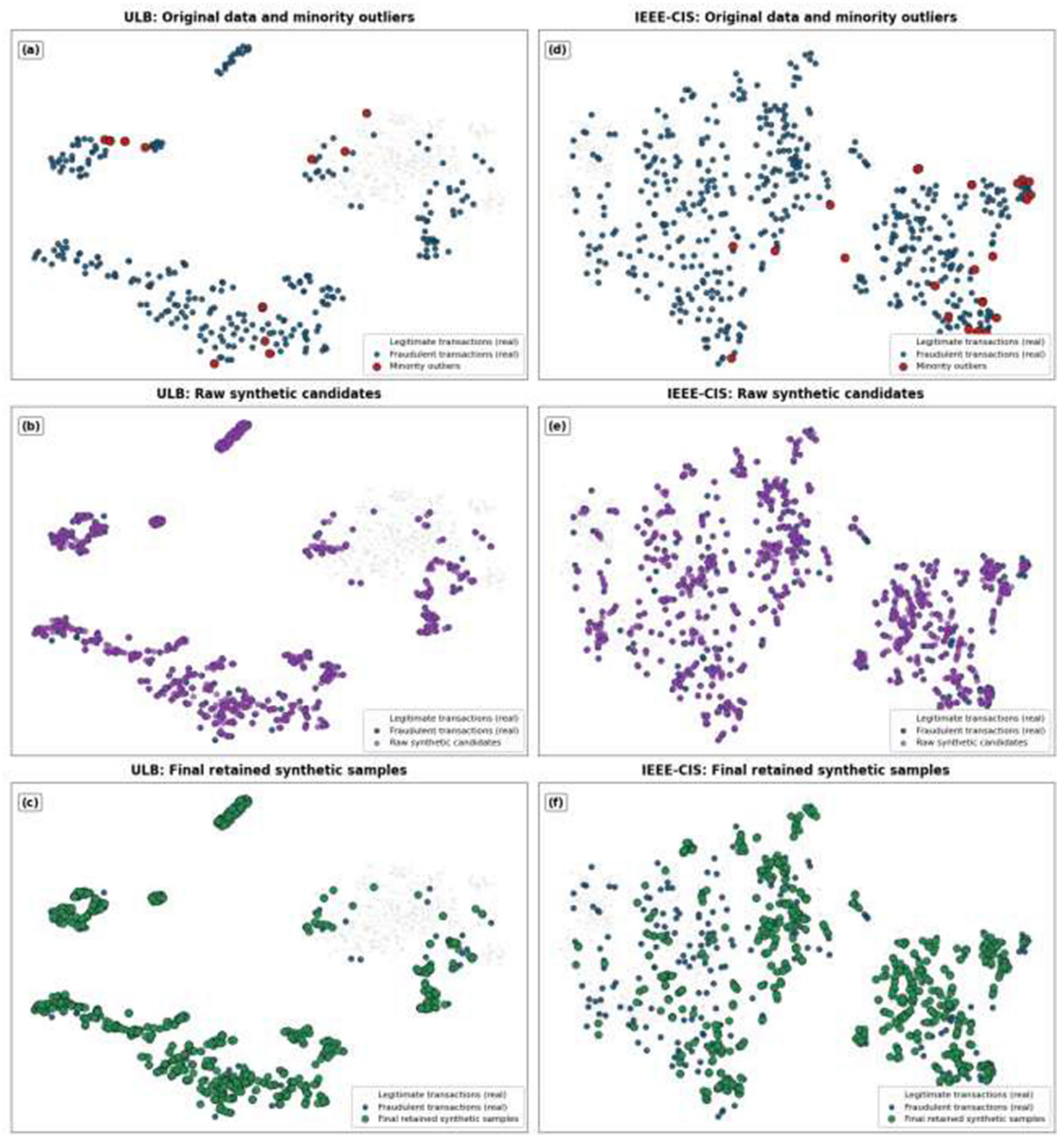
Simplified sequential visualization of the proposed method using t-SNE projection on the ULB and IEEE-CIS sets.

Figure 3 presents a simplified sequential visualization of the proposed method using two-dimensional t-SNE projection for the ULB and IEEE-CIS datasets. The visualization was generated independently for each dataset from standardized variables in the training set. To avoid visual saturation and reduce the computational cost of t-SNE, a subsample was used in each case, consisting of 500 real legitimate transactions, up to 500 real fraudulent transactions, and up to 1000 raw synthetic candidates generated using SMOTE. This subsample was used solely to graphically represent the method's flow, while the training and evaluation of the models followed the complete experimental protocol described in the corresponding sections.

The figure summarizes three key steps in the procedure for both datasets: identification of minority outliers in the real training data, initial generation of synthetic candidates using SMOTE, and final selection of retained synthetic samples after geometric and supervised plausibility filtering. The final synthetics correspond to artificial samples that simultaneously satisfy criteria of structural proximity to the minority class and discriminative consistency, according to an auxiliary classifier trained solely on real data from the training set. Euclidean distance was used for nearest neighbor calculations and the t-SNE projection. The t-SNE hyperparameters were: n_components = 2, perplexity = 50 or adjusted according to sample size, learning_rate = auto, init = pca, max_iter = 1500, and random_state = 42. This projection was used solely for exploratory purposes and not as quantitative evidence of separability between classes.

### 2.4 Method calibration

This stage was designed to evaluate the method's sensitivity to controlled changes in the parameters governing synthesis intensity and the severity of structural filters. The following variables were analyzed: the synthesis multiplier α, the outlier removal quantile *q*_*out*_, the geometric filter quantile *q*_*f*_, and the supervised plausibility threshold *t*_*p*_. The parameter *M* was maintained as a computational safety margin to prevent excessive expansion of the training set.

Internal calibration of the method was performed on the ULB dataset to select a reference operating configuration before benchmarking. Stratified cross-validation (k = 3) was used on *D*_*train*_, considering two complementary classifiers: Logistic Regression and XGBoost. AUPRC and MCC were used as selection criteria, and the final ranking was determined by an overall score based on the average of the ranks obtained in both metrics.

Instead of conducting an exhaustive search, a reduced set of configurations, presented in Table 1, was defined to analyze the method's sensitivity to controlled changes in synthesis intensity, outlier removal, geometric filtering, and plausibility threshold. The results of this calibration are presented in Table 2. Although configuration C5 achieved the best overall balance between AUPRC and MCC, configuration C0 showed very close performance and was selected as the reference configuration due to its less restrictive nature and direct comparability with ablation analysis. This configuration was kept constant in subsequent experiments, including the evaluation on IEEE-CIS, to analyze the method's transferability without specific recalibration per dataset.

**Table 1:** Configurations evaluated for internal calibration of the proposed method.

Config.	α	q_out_	q_f_	t_p_	Description
C0	2	0.95	0.75	0.75	Moderate synthesis intensity
C1	1	0.95	0.75	0.75	Lower synthesis intensity
C2	3	0.95	0.75	0.75	Higher synthesis intensity
C3	2	0.90	0.75	0.75	Stricter outlier removal
C4	2	0.99	0.75	0.75	More lenient outlier removal
C5	2	0.95	0.60	0.75	More restrictive geometric filter
C6	2	0.95	0.90	0.75	More lenient geometric filter
C7	2	0.95	0.75	0.65	Less strict plausibility
C8	2	0.95	0.75	0.85	More strict plausibility

**Table 2:** Results obtained from the calibrations performed on the proposed strategy.

Config.	Description	Mean AUPRC	Mean MCC	Global Rank Score
C5	More restrictive geometric filter	0.8092	0.8058	3
C8	More strict plausibility threshold	0.8092	0.8042	4
C4	More lenient outlier removal	0.8110	0.8036	4.5
C0	Current reference configuration	0.8088	0.8045	4.5
C3	Stricter outlier removal	0.8084	0.8049	4.5
C6	More permissive geometric filter	0.8060	0.8071	5
C2	Higher synthesis intensity	0.8052	0.8100	5
C7	Less strict plausibility threshold	0.8064	0.8039	7
C1	Lower synthesis intensity	0.8083	0.7897	7.5

### 2.5 Ablation of the proposed strategy

Ablation analysis was performed on the ULB ensemble to identify the incremental contribution of the principal components of the proposed scheme. Three variants were compared: a conservative SMOTE generation without filters, a variant with geometric filtering, and a complete variant incorporating geometric filtering and supervised plausibility. This analysis allowed us to assess whether the observed improvements were solely due to the increased number of synthetic samples or to the combined effect of the control mechanisms incorporated in the proposal. The results are presented in Table 3.

**Table 3:** Ablation analysis of the principal components of the proposed strategy.

Classifier	Variant	AUPRC	MCC	FN	FP
DecisionTree	SMOTE conservative without filters	0.4213	0.6466	19	73
DecisionTree	+ geometric filter	0.4159	0.6423	19	75
DecisionTree	+ plausibility	0.7631	0.8245	18	16
ExtraTrees	SMOTE conservative without filters	0.8538	0.8828	16	6
ExtraTrees	+ geometric filter	0.8720	0.8658	14	12
ExtraTrees	+ plausibility	0.8741	0.8221	13	24
HistGBM	SMOTE conservative without filters	0.8127	0.7659	17	33
HistGBM	+ geometric filter	0.7843	0.7701	18	30
HistGBM	+ plausibility	0.8037	0.7649	15	37
LogisticRegression	SMOTE conservative without filters	0.7193	0.8212	20	14
LogisticRegression	+ geometric filter	0.7196	0.8212	20	14
LogisticRegression	+ plausibility	0.7392	0.7621	16	36
RandomForest	SMOTE conservative without filters	0.8633	0.8451	16	14
RandomForest	+ geometric filter	0.8593	0.8408	16	15
RandomForest	+ plausibility	0.8650	0.8062	12	30
XGBoost	SMOTE conservative without filters	0.8305	0.8623	17	9
XGBoost	+ geometric filter	0.8359	0.8581	22	4
XGBoost	+ plausibility	0.8657	0.8587	16	11

Within the ablation framework, the full variant achieved the best AUPRC in five of the six evaluated classifiers. This result does not imply universal dominance across all scenarios, but it does demonstrate that supervised plausibility filtering was the component that contributed most significantly to the proposed architecture. The IEEE-CIS evaluation was subsequently performed using the final method configuration, with the aim of analyzing its performance on a second dataset without recalibrating the strategy components.

### 2.6 Comparative models

To evaluate the usefulness of the proposed method under different inductive biases, a set of representative classifiers for unbalanced tabular data was considered. Logistic regression (LR) was included as an interpretable and stable baseline, useful for verifying whether retained synthetic information improves overall separability in a parametric model. Decision Trees (DT), Random Forests (RF), and Extra Trees (ExT) allow for analyzing the method's behavior with hierarchical structures and nonlinear partitions of the attribute space. Finally, HistGradientBoostingClassifier (HGB) and XGBoost (XGB) were incorporated due to their recognized performance in tabular problems, their ability to model complex interactions, and their frequent use in fraud scenarios with severe imbalance (Mienye and Jere, 2024; Breiman, 2001; Chen and Guestrin, 2016).

To isolate the effect of the augmentation scheme, the classifiers were trained using the same partitioning, preprocessing, calibration, and threshold adjustment protocol. The proposed method was also compared against a scheme without augmentation and against conventional oversampling techniques, specifically SMOTE, ADASYN, and Borderline-SMOTE, due to their widespread adoption as benchmarks in unbalanced classification (Chawla et al., 2002; He et al., 2008; Han et al., 2005). Additionally, a generative scheme based on a variational autoencoder, called SimpleVAE, was included. This scheme had a latent dimension of 8, a hidden layer of 64 units, an Adam optimizer with a learning rate of 1 × 10^−3^, a batch size of 64, and 150 training epochs. This comparison allowed us to evaluate whether synthesis filtered by geometric coherence and supervised plausibility offers advantages over classical oversampling methods and over a simple deep generator.

For each model, hyperparameters were defined with a conservative approach, prioritizing stability, comparability between augmentation schemes, and consistency in evaluation, rather than exhaustive optimization specific to each classifier. The final hyperparameters were established based on controlled preliminary tests on the development set and are summarized in Table 4.

**Table 4:** Hyperparameters used for each classifier.

Classifier	Hyperparameters	Justification
LR	penalty = l2, C = 1.0, solver = liblinear, class_weight = balanced, max_iter = 3,000	It was used as a linear, interpretable, and robust baseline to verify whether augmentation improves global separability in a parametric model.
DT	max_depth = 8, min_samples_leaf = 5, class_weight = balanced	It allows evaluating the behavior of the method in the face of non-linear boundaries and hierarchical decision rules with controlled complexity.
RF	n_estimators = 500, max_depth = None, min_samples_leaf = 2, class_weight = balanced_subsample	It was included because of its ability to reduce variance, model nonlinear interactions, and maintain good performance on unbalanced tabular data.
ExT	n_estimators = 500, max_depth = None, min_samples_leaf = 2, class_weight = balanced_subsample	It introduces greater randomization than Random Forest, which allows testing the stability of the proposed method against highly diversified ensembles.
HGB	learning_rate = 0.03, max_depth = 6, max_iter = 500, min_samples_leaf = 20, l2_regularization = 1e-3	It was used as an efficient boosting method for tabular data, capable of modeling complex interactions and nonlinear relationships with explicit regularization.
XGB	n_estimators = 600, max_depth = 5, learning_rate = 0.03, subsample = 0.90, colsample_bytree = 0.90, reg_lambda = 1.0, objective = binary:logistic, eval_metric = aucpr, tree_method = hist, scale_pos_weight = n_neg/n_pos	It was selected for its competitive performance in unbalanced tabular classification and for allowing explicit adjustment to the imbalance using scale_pos_weight.

### 2.7 Evaluation metrics

In credit card fraud detection, evaluation should not be based solely on overall accuracy, as the marked asymmetry between classes can lead to misleading interpretations of performance. In this context, the primary focus is on the model's ability to identify the minority class without excessively degrading the accuracy of the generated alerts. Therefore, the evaluation protocol centered on metrics robust to imbalance, prioritizing precision, recall, F1-score, Matthews Correlation Coefficient (MCC), and Area Under the Precision-Recall Curve (AUPRC), with accuracy as a complementary metric (Chicco and Jurman, 2020; Saito and Rehmsmeier, 2015; Davis and Goadrich, 2006).

Let the confusion matrix be defined by the counts of true positives (*TP*), true negatives (*TN*), false positives (*FP*), and false negatives (*FN*). In the context of credit card fraud detection, a TP represents a fraudulent transaction correctly identified as such, while a TN corresponds to a legitimate transaction correctly classified as non-fraudulent. A FP occurs when a legitimate transaction is erroneously flagged as fraudulent, which can increase manual review workload, operating costs, and customer inconvenience. Conversely, an FN represents a fraudulent transaction that is not detected by the system, which can result in direct financial losses and a delayed institutional response. From these terms, accuracy, precision, and recall were calculated according to Equations 12–14, respectively:


$$\begin{array}{l} Accuracy\;=\;\frac{TP\;+TN}{TP+TN+FP+FN} \end{array}$$ (12)

$$\begin{array}{l} Precision\;=\;\frac{TP}{TP+FP} \end{array}$$ (13)

$$\begin{array}{l} Recall\;=\;\frac{TP}{TP+FN} \end{array}$$ (14)

Accuracy measures the overall performance of the classifier. Precision measures the proportion of transactions predicted as fraudulent that actually are, while recall quantifies the system's ability to recover real fraudulent events. In scenarios of severe imbalance, both metrics are more informative than accuracy, as they explicitly assess behavior toward the minority class. From these two measurements, the F1-score was calculated according to Equation 15, defined as the harmonic mean between precision and recall:


$$\begin{array}{l} F1-score\;=\;2*\frac{Precision\;*Recall}{Precision\;+\;Recall} \end{array}$$ (15)

This metric summarizes the trade-off between detection and purity of alerts, being useful when you want to avoid an improvement in recall occurring at the cost of excessive deterioration in accuracy, or vice versa.

The MCC was used as the primary measure for adjusting the decision threshold, since it simultaneously incorporates the four elements of the confusion matrix and offers a balanced assessment even under extremely skewed class distributions. Its expression in Equation 16:


$$\begin{array}{l} MCC\;=\;\frac{TP\cdot TN-FP\cdot FN}{\sqrt{\left(TP+FP\right)\left(TP+FN\right)\left(TN+FP\right)\left(TN+FN\right)}} \end{array}$$ (16)

Furthermore, the AUPRC was prioritized because the precision-recall curve is more sensitive than the ROC curve, specifically when there is an extreme data imbalance, as in the context of this study. The PR curve represents the combined behavior of precision and recall for different decision thresholds, and its area under the curve is expressed in Equation 17:


$$\begin{array}{l} AUPRC\;=\;\int_{0}^{1}Precision\left(r\right)dr, \end{array}$$ (17)

where *r* represents the recall. A higher AUPRC indicates that the classifier maintains good accuracy across different sensitivity levels, which is particularly relevant in anti-fraud applications where an excess of false positives can compromise the operational utility of the system.

## 3 Results

The proposed data augmentation process preserved the unbalanced nature of both training sets while selectively enriching the minority class. As shown in Table 5, ULB started with 284 actual fraudulent transactions, of which 269 were retained as the basis for synthetic generation after excluding 15 minority observations considered local outliers. From this subset, 852 raw synthetic samples were generated using SMOTE; subsequently, the geometric filter retained 846 candidates, and the supervised plausibility filter reduced the final set to 638 synthetics. This increased the fraudulent class from 284 to 922 observations, and the final fraud rate reached 0.005396.

**Table 5:** Summary of the proposed augmentation process on the training set using the proposed method.

Stage	ULB	IEEE-CIS
Majority samples	169,951	341,926
Original minority samples	284	12,398
Minority inliers retained for SMOTE	269	11,778
Minority outliers removed	15	620
Generated raw synthetics	852	5,000
After geometric filter	846	4,674
After plausibility filter	638	947
Final synthetics retained	638	947
Final fraud rate after augmentation	0.005396	0.037563

At IEEE-CIS, the procedure followed the same conservative logic. Of 12,398 actual fraudulent transactions, 11,778 minority instances were retained for SMOTE after removing 620 local outliers. The initial generation produced 5,000 synthetic candidates, of which 4,674 passed the geometric filter and 947 were retained after the supervised plausibility filter. Thus, the minority class increased from 12,398 to 13,345 observations, with a final fraud rate of 0.03756. In both cases, the final fraud rate was calculated as the ratio between the total number of minority samples, considering the original fraudulent transactions and the final retained synthetics, and the total number of samples in the augmented training set.

Tables 6, 7 present the final distribution of the training sets for ULB and IEEE-CIS according to the augmentation strategy applied. In both sets, the conventional methods SMOTE and Borderline-SMOTE generated practically balanced distributions, with a final fraud rate close to 0.50, while ADASYN produced similar results. SimpleVAE increased the minority class more moderately, adding 852 synthetic samples in ULB and 5,000 in IEEE-CIS. The proposed strategy maintained an even more conservative approach: in ULB it incorporated 638 synthetics, increasing the minority class from 284 to 922 observations, while in IEEE-CIS it added 947 synthetics, increasing the fraudulent class from 12,398 to 13,345 cases.

**Table 6:** Final distribution of the training set according to the augmentation strategy in ULB.

Augmentation method	Total samples	Majority (Class 0)	Minority (Class 1)	Synthetics added	Final fraud rate
NoAug	170,235	169,951	284	0	0.001668
SMOTE	339,902	169,951	169,951	169,667	0.5
Borderline-SMOTE	339,902	169,951	169,951	169,667	0.5
ADASYN	339,880	169,951	169,929	169,645	0.499968
SimpleVAE	171,087	169,951	1,136	852	0.00664
Proposed strategy	170,873	169,951	922	638	0.005396

**Table 7:** Final distribution of the training set according to the augmentation strategy in IEEE-CIS.

Augmentation method	Total samples	Majority (Class 0)	Minority (Class 1)	Synthetics added	Final fraud rate
NoAug	354,324	341,926	12,398	0	0.034991
SMOTE	683,852	341,926	341,926	329,528	0.5
Borderline-SMOTE	683,852	341,926	341,926	329,528	0.5
ADASYN	681,780	341,926	339,854	327,456	0.49848
SimpleVAE	359,324	341,926	17,398	5,000	0.048419
Proposed strategy	355,271	341,926	13,345	947	0.037563

**Figure 4:**
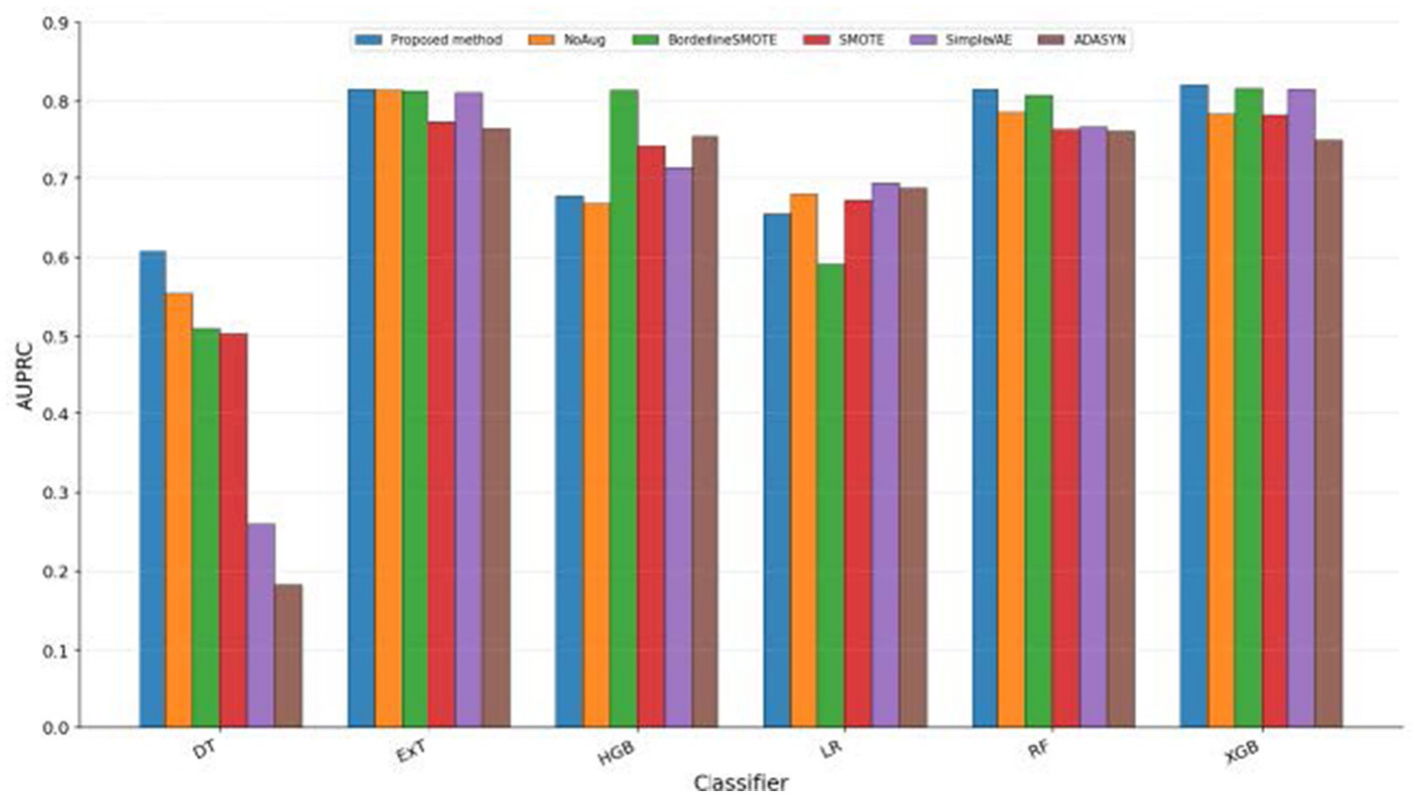
AUPRC comparison across classifiers and augmentation strategies in ULB.

After characterizing how each strategy modified the distribution of the training set, its impact on the predictive performance of the classifiers was evaluated. For this purpose, the AUPRC was used as the main comparison metric, as it allows for a more detailed analysis of the model's behavior with respect to the fraudulent class in a context of extreme imbalance. In this type of problem, a metric focused on the relationship between precision and recall offers a more informative reading than overall accuracy. Figure 4 shows in greater detail the variation in the performance of each augmentation strategy according to the classifier used on the ULB set. The proposed method showed outstanding performance in tree-based and ensemble classifiers. It achieved the highest AUPRC in DT (0.606955), ExT (0.815339), RF (0.815723), and XGB (0.820714). In contrast, for HGB the best result was obtained with BorderlineSMOTE (0.814642), and for LR with SimpleVAE (0.693720).

**Figure 5:**
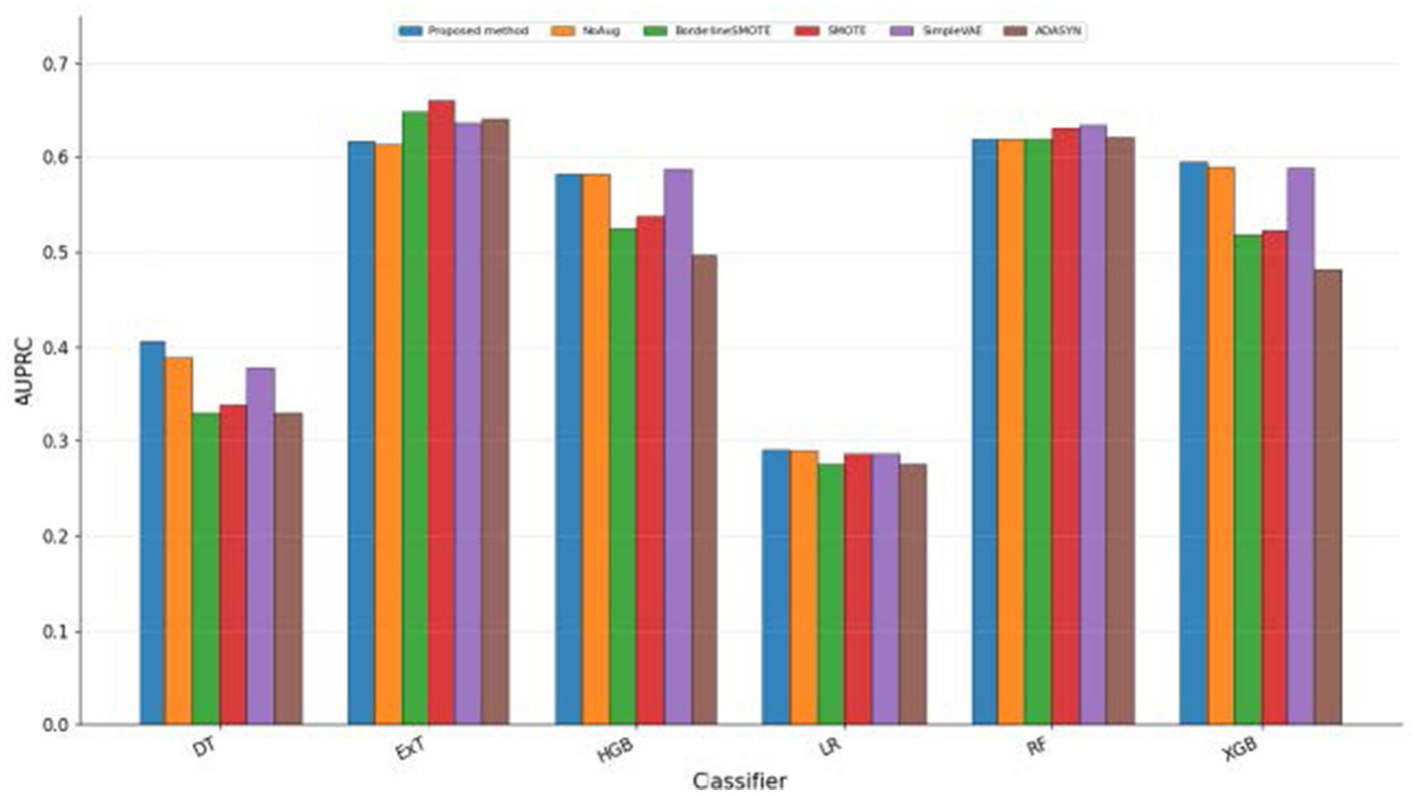
AUPRC comparison across classifiers and augmentation strategies in IEEE-CIS.

In IEEE-CIS (Figure 5), the strategies' performance was less uniform than in ULB. The proposed method achieved the highest AUPRC in DT (0.406319), LR (0.290234), and XGB (0.595169). In the other classifiers, the best performance was obtained with other strategies: SMOTE in ExT (0.661974), SimpleVAE in HGB (0.587109), and SimpleVAE in RF (0.636156). This variation indicates that the usefulness of each augmentation strategy changes depending on the classifier and the dataset structure. Even so, the proposed strategy maintained competitive performance in the second dataset, providing further evidence of its transferability, although without assuming uniform superiority in all evaluated scenarios.

The difference between accuracy and positive class metrics stems from the fact that accuracy is heavily influenced by the large number of legitimate transactions correctly classified. Therefore, some errors in fraud detection barely affect overall accuracy, but they do reduce recall and precision, since these metrics directly assess the ability to detect actual fraud and the reliability of the generated alerts.

Table 8 presents the best results obtained in the test set for each classifier in the ULB dataset. Although all models achieved accuracy values greater than 0.999, this metric should be interpreted with caution due to the extreme imbalance of the dataset. Therefore, the analysis also considered precision, recall, F1-score, MCC, and classification errors. HGB with BorderlineSMOTE obtained the highest F1-score (0.85549), MCC (0.85945), and precision (0.948718), with only 4 false positives and 21 false negatives. XGB with the proposed method presented one of the best overall balances, with a precision of 0.892857, recall of 0.78947, F1-score of 0.83799, MCC of 0.83933, 9 false positives, and 20 false negatives. RF with the proposed method showed very similar behavior, with the same recall (0.78947), F1-score of 0.82873 and MCC of 0.82949. The proposed method was the best configuration for DT, ExT, RF, and XGB, while HGB achieved its best result with BorderlineSMOTE and LR with SimpleVAE.

**Table 8:** Best test result by classifier among the augmentation strategies evaluated in the ULB set.

Classifier	Augmentation strategy	Accuracy	Precision	Recall	F1	MCC	TN	FP	FN	TP
DT	Proposed method	0.99924	0.851351	0.66316	0.74556	0.75103	56640	11	32	63
ExT	Proposed method	0.99944	0.870588	0.77895	0.82222	0.82322	56640	11	21	74
HGB	BorderlineSMOTE	0.99956	0.948718	0.77895	0.85549	0.85945	56647	4	21	74
LR	SimpleVAE	0.99938	0.857143	0.7579	0.80447	0.80569	56639	12	23	72
RF	Proposed method	0.99945	0.872093	0.78947	0.82873	0.82949	56640	11	20	75
XGB	Proposed method	0.99949	0.892857	0.78947	0.83799	0.83933	56642	9	20	75

Table 9 presents the best results obtained in the IEEE-CIS dataset. Unlike ULB, accuracy values were lower, and the metrics associated with the fraudulent class showed greater variability, reflecting the greater complexity of the dataset. In this scenario, ExT with SMOTE achieved the highest F1-score (0.640225), MCC (0.638383), and recall (0.550690), with 701 false positives and 1857 false negatives. RF with SimpleVAE also showed competitive performance, with an F1-score of 0.605671, MCC of 0.601334, and recall of 0.524558. The proposed method was the best configuration for DT, LR, and XGB. In particular, XGB with the proposed method achieved a precision of 0.760210, recall of 0.454875, F1-score of 0.569180, and MCC of 0.577135, with 593 false positives and 2253 false negatives.

**Table 9:** Best test result by classifier among the augmentation strategies evaluated in the IEEE-CIS set.

Classifier	Augmentation strategy	Accuracy	Precision	Recall	F1	MCC	TN	FP	FN	TP
DT	Proposed method	0.969011	0.591419	0.370191	0.455357	0.453123	112918	1057	2603	1530
ExT	SMOTE	0.978342	0.764528	0.55069	0.640225	0.638383	113274	701	1857	2276
HGB	SimpleVAE	0.975649	0.748517	0.458021	0.568298	0.574361	113339	636	2240	1893
LR	Proposed method	0.947548	0.306567	0.395354	0.345345	0.321277	110279	3696	2499	1634
RF	SimpleVAE	0.976098	0.716457	0.524558	0.605671	0.601334	113117	858	1965	2168
XGB	Proposed method	0.975903	0.76021	0.454875	0.56918	0.577135	113382	593	2253	1880

**Figure 6:**
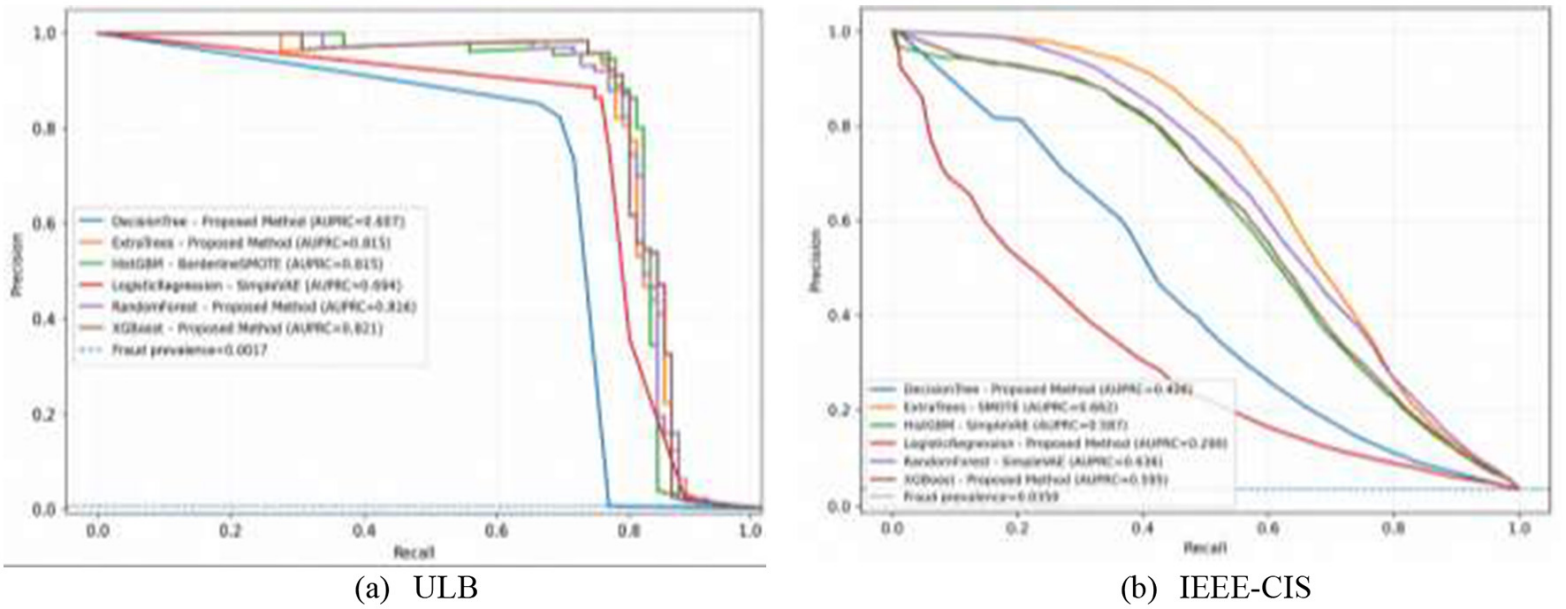
Precision-Recall curves for the best-performing configuration of each classifier in ULB and IEEE-CIS.

To complement the tabular comparison described above, Figure 6 present the precision-recall curves corresponding to the best-performing scenario for each classifier on the ULB and IEEE-CIS datasets. This visualization allowed for a more direct comparison of model behavior under extreme class imbalance, as the precision-recall curve centers on the positive class, which corresponds to fraudulent transactions. The horizontal dashed line represents the prevalence of fraud in the test set and serves as a baseline. Models with curves higher than this baseline and higher AUPRC values offer better discrimination of fraudulent transactions at different levels of exhaustiveness.

Finally, statistical validation based on stratified bootstrap resampling and McNemar tests for paired errors was incorporated. Table 10 presents the bootstrap estimates and 95% confidence intervals for the best-performing configuration of each classifier, selected according to the AUPRC in the ULB ensemble. The proposed method achieved the best AUPRC in four of the six classifiers: XGB, RF, ExT, and DT. The highest AUPRC was obtained with XGB using the proposed method, reaching 0.821 with a 95% confidence interval of 0.740–0.888. RF and ExT with the proposed method showed similar AUPRC values of 0.816 and 0.815, respectively, with overlapping confidence intervals. This indicates that these ensemble-based classifiers exhibited comparable and stable behavior under bootstrap resampling. Regarding the classifier, HGB achieved its best configuration with BorderlineSMOTE, while LR performed better with SimpleVAE, suggesting that the advantage of the proposed augmentation strategy depends on the classifier and is not universal.

**Table 10:** Bootstrap summary for the best configuration of each classifier in dataset ULB.

Classifier	Augmentation strategy	AUPRC	MCC	F1-score	Precision	Recall
XGB	Proposed method	0.821 (0.740, 0.888)	0.839 (0.777, 0.891)	0.838 (0.774, 0.890)	0.893 (0.824, 0.959)	0.789 (0.705, 0.863)
RF	Proposed method	0.816 (0.735, 0.885)	0.829 (0.767, 0.883)	0.829 (0.767, 0.883)	0.872 (0.806, 0.938)	0.789 (0.705, 0.863)
ExT	Proposed method	0.815 (0.733, 0.885)	0.823 (0.761, 0.882)	0.822 (0.756, 0.881)	0.871 (0.802, 0.938)	0.779 (0.695, 0.853)
HGB	BorderlineSMOTE	0.815 (0.731, 0.889)	0.859 (0.803, 0.912)	0.855 (0.795, 0.908)	0.949 (0.897, 0.988)	0.779 (0.695, 0.863)
LR	SimpleVAE	0.694 (0.598, 0.792)	0.806 (0.744, 0.865)	0.804 (0.741, 0.863)	0.857 (0.787, 0.925)	0.758 (0.663, 0.832)
DT	Proposed method	0.607 (0.501, 0.713)	0.751 (0.679, 0.821)	0.746 (0.671, 0.816)	0.851 (0.776, 0.925)	0.663 (0.558, 0.758)

McNemar's analysis in Table 11 provided a paired-error comparison between the proposed method and the baseline augmentation strategies. After Holm-Bonferroni correction, statistically significant differences were observed in three comparisons. For DT, the proposed method showed significant improvements in paired errors compared to SimpleVAE and ADASYN. In both cases, the number of instances corrected by the proposed method was substantially higher than the number of misclassified instances relative to the baseline, with 128 vs. 12 for SimpleVAE and 224 vs. 14 for ADASYN. A significant difference was also observed for LR compared to BorderlineSMOTE, where the proposed method corrected 23 baseline errors while introducing only 3 new errors. For the remaining comparisons between classifiers and reference models, McNemar's test showed no statistically significant differences after correction, indicating that the proposed method achieved competitive performance but was not uniformly superior across all scenarios.

**Table 11:** Representative paired comparisons using McNemar between the proposed method and reference strategies in ULB.

Classifier	Augmentation strategy	Baseline	Baseline correct/proposed wrong	Baseline wrong/proposed correct	McNemar statistic	*p*-value	Holm-adjusted *p*-value	Interpretation
DT	Proposed method	SimpleVAE	12	128	94.464	< 0.001	< 0.001	Significant
DT	Proposed method	ADASYN	14	224	183.534	< 0.001	< 0.001	Significant
LR	Proposed method	BorderlineSMOTE	3	23	13.885	< 0.001	0.005	Significant

Statistical validation for IEEE-CIS is presented in Tables 12, 13. In Table 12, the bootstrap summary shows that the best AUPRC was obtained by ExT with SMOTE, with 0.662 (0.648, 0.676), followed by RF with SimpleVAE, with 0.636 (0.623, 0.650), and XGB with the proposed method, with 0.595 (0.580, 0.611). The proposed method was the best configuration for XGB, DT, and LR, while SMOTE and SimpleVAE achieved better results for ExT, HGB, and RF. Table 13 summarizes three representative paired comparisons using McNemar's test after Holm-Bonferroni correction. In XGB and HGB, the proposed method corrected more errors than BorderlineSMOTE, with 657 vs. 293 and 834 vs. 415 discordant cases, respectively. In ExT, the comparison against SMOTE favored the reference strategy, with 411 cases correctly classified by SMOTE and failed by the proposed method, compared to 190 cases corrected by the proposed method. These findings show that, in IEEE-CIS, the proposed method maintained competitive performance and statistically significant differences in key comparisons, although its advantage depended on the classifier used.

**Table 12:** Bootstrap summary for the best configuration of each classifier in IEEE-CIS dataset.

Classifier	Augmentation strategy	AUPRC	MCC	F1-score	Precision	Recall
ExT	SMOTE	0.662 (0.648, 0.676)	0.638 (0.626, 0.651)	0.640 (0.627, 0.653)	0.765 (0.751, 0.778)	0.551 (0.535, 0.566)
RF	SimpleVAE	0.636 (0.623, 0.650)	0.601 (0.589, 0.614)	0.606 (0.593, 0.619)	0.716 (0.702, 0.732)	0.525 (0.510, 0.540)
XGB	Proposed method	0.595 (0.580, 0.611)	0.577 (0.564, 0.592)	0.569 (0.556, 0.584)	0.760 (0.745, 0.776)	0.455 (0.441, 0.471)
HGB	SimpleVAE	0.587 (0.573, 0.602)	0.574 (0.561, 0.589)	0.568 (0.555, 0.583)	0.749 (0.733, 0.765)	0.458 (0.444, 0.474)
DT	Proposed method	0.406 (0.392, 0.423)	0.453 (0.439, 0.467)	0.455 (0.441, 0.470)	0.591 (0.576, 0.610)	0.370 (0.355, 0.385)
LR	Proposed method	0.290 (0.277, 0.304)	0.321 (0.309, 0.334)	0.345 (0.334, 0.357)	0.307 (0.296, 0.318)	0.395 (0.380, 0.410)

**Table 13:** Representative paired comparisons using McNemar between the proposed method and reference strategies in IEEE-CIS.

Classifier	Augmentation	Baseline	Baseline correct/ proposed wrong	Baseline wrong/ proposed correct	McNemar statistic	Holm-adjusted *p*-value	Interpretation
XGB	Proposed method	BorderlineSMOTE	293	657	138.704	< 0.001	Significant
HGB	Proposed method	BorderlineSMOTE	415	834	139.891	< 0.001	Significant
ExT	Proposed method	SMOTE	411	190	80.532	< 0.001	Significant

These statistical results lead to a more cautious interpretation of the proposed method. In several scenarios, the proposed method improved upon or matched the most competitive configurations across different classifiers and was particularly effective with tree-based ensemble models. However, statistical validation also shows that its benefit depends on the classifier and reference model considered for both datasets.

## 4 Discussion

The results obtained support the hypothesis that, in fraud detection with extreme imbalance, the benefit does not come from indiscriminately increasing the minority class, but rather from controlling which synthetic samples are incorporated into the training. The proposed method combines local generation using SMOTE with two mechanisms: geometric filtering and supervised plausibility. This approach prevents the augmentation of minority class data from functioning as a massive expansion. Validation using bootstrap and McNemar supports this interpretation, as it showed competitive performance and significant differences in specific comparisons; however, it also demonstrated that the benefit of the method varies depending on the classifier and the dataset being evaluated.

The comparison between ULB and IEEE-CIS shows that the method's performance depends on both the dataset structure and the classifier used. In ULB, where the imbalance is more extreme, the proposed method achieved the highest AUPRC in four of the six evaluated classifiers: DT, ExT, RF, and XGB. In IEEE-CIS, the performance was more heterogeneous, with the best AUPRC obtained in DT, LR, and XGB, while SMOTE and SimpleVAE achieved better values in other classifiers. The ablation analysis performed on ULB reinforces this interpretation, as the full variant with supervised plausibility obtained the best AUPRC in five of the six classifiers, with particularly noticeable improvements in Decision Tree and XGBoost. However, the same mechanism that provides quality control also explains the method's conservative nature: the filter favors synthetic candidates compatible with already observed fraudulent patterns, so its main contribution lies in reinforcing plausible regions of the minority class, not in discovering new forms of fraud.

Regarding the literature cited in Table 14, it has been shown that strategies such as SMOTE, ADASYN, or GNUS can offer varying benefits depending on the degree of imbalance and the decision model, but also that these approaches can introduce ambiguous or insufficiently plausible observations (Imani et al., 2025; Becerra-Suarez et al., 2025b). Similarly, more recent studies based on VAE, tabular GANs, sequential models, and comparisons between CTGAN, TVAE, or Gaussian noise maintain that there is no universally superior generator and that performance is sensitive to preprocessing, encoding, hyperparameters, and distributional preservation (Yousefimehr and Ghatee, 2025; Lee and Bae, 2025; Won et al., 2026; Kindji et al., 2025). Within this framework, the distinctive contribution of the present work lies in shifting the focus from mere generation to the structural selection of synthetics, aligning with the AGSS proposal (Nama and Banu, 2026), but adding more explicit control over supervised plausibility.

**Table 14:** Conceptual comparison of recent approaches to data augmentation and synthetic generation for imbalanced classification.

Study/dataset	Main approach	Relevant limitation or gap	Relationship to this study
(Imani et al., 2025) Customer churn datasets with different imbalance levels, ranging from 15% to 1% minority class.	Comparative evaluation of Random Forest and XGBoost combined with SMOTE, ADASYN, and GNUS under different imbalance levels.	The effectiveness of data augmentation depends on the classifier, the oversampling technique, and the degree of imbalance. SMOTE was more effective with XGBoost, ADASYN showed moderate benefits, and GNUS produced inconsistent results.	This finding supports the idea that data augmentation should not be evaluated only by the number of generated samples, but also by its interaction with the classifier. Unlike that approach, our method does not aggressively balance the minority class, but selects synthetic samples based on geometric coherence and supervised plausibility.
(Becerra-Suarez et al., 2025b) ULB Credit Card Fraud Detection dataset: European card transactions with 492 fraudulent cases and severe class imbalance.	Data augmentation through controlled Gaussian noise for bank fraud detection, compared against SMOTE and ADASYN using machine learning and deep learning classifiers.	The method depends on the calibration of the noise parameter (α) and does not explicitly filter the geometric coherence or discriminative plausibility of each synthetic sample before adding it to the training set.	This study preserves the goal of avoiding redundant synthetic samples but shifts the focus from global perturbation to post-generation selection through explicit filters.
(Yousefimehr and Ghatee, 2025) European credit card fraud dataset with sequential transaction analysis.	Distribution-preserving resampling combined with OCSVM, SMOTE, undersampling, LightGBM, and LSTM for sequential and non-sequential fraud detection.	The approach involves greater methodological complexity due to the combination of several components: distribution preservation, outlier detection, window selection, and sequential models. It does not filter each synthetic sample through individual supervised plausibility.	This work supports the idea that augmentation should preserve relevant minority-class information. Our approach pursues a similar objective through a simpler route: conservative SMOTE, geometric filtering, and supervised plausibility filtering.
(Lee and Bae, 2025) Five public tabular datasets: SECOM, CREDIT, THYROID, APS, and UCI.	Hybrid architecture using VAE-based augmentation, deep ensemble learning, and models for imbalanced tabular classification.	The effectiveness of augmentation is dataset-dependent; VAE does not dominate universally, and in some settings SMOTE or even no augmentation can perform better. The approach also entails greater architectural complexity.	This reinforces the idea that there is no universally superior augmentation technique. Our proposal adopts a simpler and more auditable alternative based on geometric selection and supervised plausibility.
(Won et al., 2026) UCI Bank Marketing dataset for imbalanced tabular classification.	Comparison of synthetic data generation methods: SMOTE, Gaussian Copula, TVAE, and CTGAN.	Statistical fidelity, predictive utility, and stability are not always maximized by the same generator. Deep generative models require greater tuning and fidelity control.	This aligns with our argument that synthetic data quality should not be inferred solely from the generator used. Our proposal filters synthetic samples before incorporating them into the training set.
(Kindji et al., 2025) Benchmark of 16 diverse tabular datasets, with an average size of approximately 80,000 records.	Broad benchmark of tabular generative models evaluated in terms of realism, utility, anonymity, computational cost, and extensive tuning.	Tabular generative models are highly sensitive to preprocessing, encoding choices, hyperparameters, and training budget. No model is clearly superior across all datasets.	Our proposal addresses this limitation through a low-cost conservative strategy, without training deep generative models or requiring extensive architectural tuning.
(Nama and Banu, 2026) Kaggle Credit Card Fraud dataset and German Credit Risk dataset.	AGSS, an adaptive oversampling method based on DBSCAN clustering, integrated with LSTM, RNN, GAN, and Transformer encoder architectures.	Although AGSS reduces noise and better preserves the minority distribution, its integration with multiple deep architectures increases methodological and computational complexity. Its control mechanism is based on density or clustering, not on individual supervised plausibility.	This is one of the closest antecedents because it also avoids indiscriminate synthetic generation. Our contribution differs by using a more parsimonious strategy: conservative SMOTE, geometric filtering, and supervised plausibility filtering.
This study ULB and IEEE-CIS	Conservative SMOTE with geometric filtering and supervised plausibility filtering for credit card fraud detection under extreme class imbalance.	The method does not aim to solve the problem through massive rebalancing or through an independent deep generative model. Its scope is focused on selecting useful synthetic samples generated from SMOTE.	It provides a parsimonious and auditable strategy that prioritizes the discriminative quality of synthetic samples over their volume. The ablation analysis showed that supervised plausibility was the most influential component.

The proposed approach is closer to those that view augmentation as a form of structural regularization than to those that understand it as simple class expansion. This observation is consistent with the discussion by Won et al. (2026); Kindji et al. (2025), and Nama and Banu (2026) and is supported by the fact that the proposed method improves AUPRC in several classifiers without incurring extreme rebalancing.

The main strength of this approach is that it combines parsimony, procedural interpretability, and relative efficiency. It is parsimonious because, unlike classical schemes that generated approximately 170,000 synthetic samples and artificially raised the fraud rate to 50%, this proposal added only 638 and 947 synthetic data points to the ULB and IEEE-CIS datasets, respectively. It is procedurally interpretable because each retained synthetic passed explicit filters for geometric consistency and supervised plausibility. Finally, it is efficient because it preserves a limited expansion of the training set without sacrificing predictive competitiveness. Therefore, the main contribution lies not in a universal dominance of metrics, but in offering a conservative alternative that prioritizes the discriminative utility of synthetics over their volume.

Despite these contributions, the study has limitations that must be acknowledged. Although two datasets were included, both fall within the domain of financial fraud and do not cover the full range of real-world transactional scenarios. Furthermore, the experimental protocol controlled for information leakage through separate training, calibration, and testing partitions, but did not explicitly assess temporal distribution changes or multi-cohort scenarios. Another limitation relates to the supervised plausibility filter: the auxiliary classifier was trained using real observations from the training set and then used to filter synthetic candidates generated from the same distribution. This decision avoids using calibration or test data and prevents the classifier from learning from previous synthetic samples, but it may also favor candidates similar to already observed patterns and discard novel fraudulent configurations. Finally, the evaluation focused on discriminative metrics, without incorporating explicit modeling of operating costs or decision policies dependent on the institutional context.

Future research should extend the evaluation to more datasets, incorporate temporal validation, analyze scenarios with distribution changes, and explore auxiliary filtering schemes using independent partitions or out-of-fold predictions. It would also be pertinent to integrate cost-sensitive thresholds, analyze the operational impact of false positives and false negatives, and compare the proposal with more recent tabular generators or adaptive synthetic sample selection strategies.

## 5 Conclusions

This study proposed a conservative data augmentation strategy for detecting credit card fraud under severe imbalance. The method combines synthetic generation using SMOTE with geometric filtering and supervised plausibility to select locally consistent and discriminatively useful artificial samples without imposing massive class rebalancing. In ULB, the proposal added 638 final synthetic samples and achieved the best AUPRC in four of the six evaluated classifiers. In IEEE-CIS, 947 final synthetics were incorporated, and the method achieved the best AUPRC in three classifiers, showing competitive performance in a second dataset with a more heterogeneous structure.

The results indicate that the quality of synthetic samples can be as relevant as their quantity in tabular financial fraud scenarios. Statistical validation and ablation analysis support the role of plausibility filtering as a quality control mechanism, although the method's benefit varies depending on the classifier and dataset. Therefore, the proposal should be interpreted as a parsimonious and conservative alternative for strengthening the minority class, not as a universally superior solution. Future research should evaluate the method on more datasets, incorporate temporal validation, analyze scenarios with distribution shifts, and explore auxiliary filtering schemes based on independent partitions or out-of-fold predictions.

## Data Availability

The original contributions presented in the study are included in the article/supplementary material, further inquiries can be directed to the corresponding author.
